# *Staphylococcus lugdunensis*: an unusual cause of relapsing hematogenous septic arthritis of a native knee. Case report and review of the literature

**DOI:** 10.3389/fmed.2024.1494449

**Published:** 2024-11-14

**Authors:** Alexandre-Raphael Wery, Maxime Taghavi, Joelle Nortier, Bhavna Mahadeb, Ioannis Raftakis, Evelyne Maillart, Philippe Clevenbergh

**Affiliations:** ^1^Department of Internal Medicine, Brugmann University Hospital, Université Libre de Bruxelles, Brussels, Belgium; ^2^Department of Nephrology, Brugmann University Hospital, Université Libre de Bruxelles, Brussels, Belgium; ^3^Department of Microbiology, Brugmann University Hospital, Université Libre de Bruxelles, Brussels, Belgium; ^4^Department of Rheumatology, Brugmann University Hospital, Université Libre de Bruxelles, Brussels, Belgium; ^5^Department of Infectious Diseases, Brugmann University Hospital, Université Libre de Bruxelles, Brussels, Belgium

**Keywords:** *Staphylococcus lugdunensis*, septic arthritis, bacteriemia, hemodialysis, arteriovenous fistula

## Abstract

*Staphylococcus lugdunensis* is a facultatively anaerobic gram-positive cocci of the coagulase-negative staphylococcus (CoNS) species. Initially considered as commensal, *S. lugdunensis* has been found to be responsible for a wide range of disseminated infections in humans (bacteriemia, foreign-body infection, endocarditis, arthritis, osteomyelitis, …) thereby often compared to *Staphylococcus aureus* in terms of virulence behavior. We present the case of a 62-year-old woman with end-stage renal disease, undergoing hemodialysis through an arteriovenous fistula (AVF) of the left forearm. She was diagnosed with *S. lugdunensis* bacteriemia and secondary native-knee septic arthritis. Endocarditis was ruled out and the patient evolved well with a 6-week course of IV cefazolin. Four months later, she consulted the rheumatology department with a recurrent right knee arthritis. Cultures came back positive for an identical multi-sensitive *S. lugdunensis*. Endocarditis was ruled out and full body 18F-FDG PET-CT showed no secondary location but a focal hypermetabolic activity in the left forearm fistula area. AVF Doppler showed no sign of collection nor thrombophlebitis around the fistula. We concluded in an infection of the fistula due to repeated punctures (recurrent cannulation) as entry point. She was treated with a 12-week oral combination of ciprofloxacin and trimethoprim-sulfamethoxazole, and her symptoms have not returned since. *S. lugdunensis* commonly causes prosthetic and arthroscopy-related joint infections. As native-joint septic arthritis is unusual, we conducted a review of the literature and discuss the burden of disseminated *S. lugdunensis* infections among dialysis patients.

## Introduction

*Staphylococcus lugdunensis* is a coagulase-negative staphylococcus (CoNS) species, firstly described by Jean Freney et al. (1) at the *Center National de Référence des Staphylocoques*, Lyon, France (thereby giving its name *lugdunensis*, Latin adjective of *lugdunum*, Latin name of Lyon). The strains were isolated from different parts of the human body (blood, axillary lymph node, umbilicus, intrauterine device, thoracic and abscess drain), and were described as nonsporulating, nonmotile and facultatively anaerobic gram-positive cocci, with a negative coagulase test with rabbit and bovine plasma (1).

Human-associated CoNS are mainly represented by *Staphylococcus epidermidis*-like group (including *S. epidermidis*, *S. haemolyticus*, *S. hominis*, *S. capitis*, …) which often cause foreign body-infections and related bloodstream infections as well as preterm newborn infections (2). Other CoNS species include *Staphylococcus saprophyticus* (mostly found in urinary tract infections) and *Staphylococcus lugdunensis*.

*S. lugdunensis* is physiologically found in human skin and mucosa, principally in the perineum and groin area, axilla, lower limbs and toes regions (3). Initially considered as commensal, *S. lugdunensis* has proved to play an important role in a wide range of infections in humans (4, 5). Indeed, while most of CoNS species were historically considered as low virulence pathogens, *S. lugdunensis* is considered as more aggressive and often compared to *Staphylococcus aureus* in terms of virulence behavior. It is responsible for a relatively similar spectrum of invasive and disseminated infections in humans (6, 7). This can be explained by analogous features present in both species, namely regions of homology in the accessory gene regulator locus (regulating virulence factors), heat-stable *δ*-like hemolysin (avoiding killing and digestion from phagosomes) and OatA (O-acetyltransferase) explaining its resistance capacity to lysozymes (4). However, toxins production by *S. aureus* such as enterotoxins, TSST and exfoliatin have not been observed in *S. lugdunensis*. Differentiating *S. lugdunensis* from other CoNS species seems particularly important in numerous situations, as management of staphylococcal bacteriemia differs significantly following the involved subspecies. In spite of that, many laboratories did not routinely identify CoNS subspecies, therefore its pathogenic role in humans may be underestimated (2). *S. lugdunensis* commonly causes skin and soft tissue infections, endocarditis including on native and prosthetic valves, catheter-related bacteriemia, prosthetic joint infections or arthroscopy-related joint infections, osteomyelitis and discitis (2). Foreign body-associated infections are easily explained by its adherence capacity and biofilm formation (4, 8). However, some rarer cases of native joint infections have been described.

## Case presentation

We present a 62-year-old woman diagnosed with recurrent right knee arthritis due to *S. lugdunensis*. Her medical history included end-stage renal disease (ESRD), type 2 diabetes, high blood pressure, bilateral gonarthrosis and cured B and C hepatitis. She underwent hemodialysis (HD) through a native arteriovenous fistula (AVF) of the left forearm surgically created in June 2019. AVF puncture was performed utilizing the buttonhole technique for hemodialysis.

She was admitted to the emergency department in April 2023 in a context of growing pain and swelling of her right knee for about a week, without any exterior trauma. She did not report fever nor chills over the past few days prior to the hospitalization. On physical examination, her right knee was warm and swollen, with a positive patellar tap test. Cardiac and pulmonary examinations were normal. The AVF exhibited a normal thrill, there was no pain nor sign of local inflammation.

**Figure 1:**
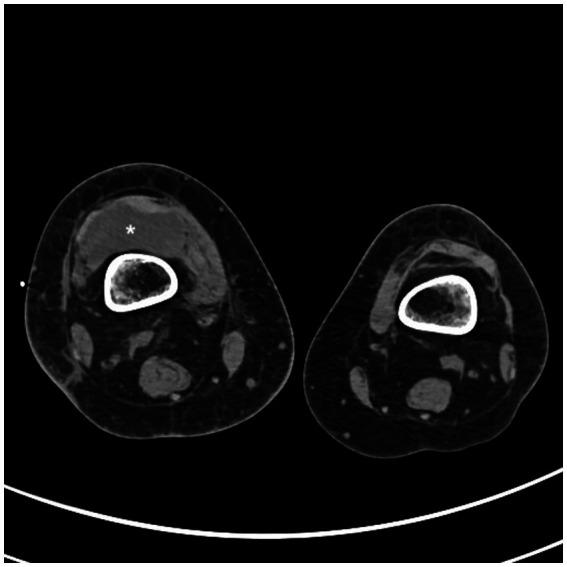
CT scan of the knees showing a voluminous intra-articular right effusion (*).

Conventional radiography of the right knee showed an irregular lateral tibial plateau. Computed tomography (CT) scan of the right knee showed a voluminous intra-articular effusion (Figure 1), internal femorotibial osteoarthritis and no sign of bone fracture. Knee ultrasound showed a large heterogeneous intra-articular compartmentalized effusion. Biology showed an elevated c-reactive protein (CRP) of 108.5 mg/L without hyperleukocytosis. Ultrasound-guided puncture of the right knee revealed 120.000 red blood cells (RBC)/μL, 91.675 white blood cells (WBC)/μL, including 99% neutrophils. Empiric antibiotherapy with IV oxacillin was initiated. Aerobic culture came back positive for *Staphylococcus lugdunensis,* which was sensitive to all the antibiotics tested (including oxacillin, clindamycin, trimethoprim-sulfamethoxazole, ciprofloxacin, linezolid, rifampicin, vancomycin). Blood cultures from the AVF came back positive for *S. lugdunensis* whereas three sets of blood cultures from peripheral veins at the same time remained negative. Cultures of repeated knee punctures to release effusion kept positive for *S. lugdunensis* for 5 days. She benefited from arthroscopic drainage and washout. Endocarditis was ruled out with transthoracic and transesophageal echocardiograms. AVF Doppler showed fibrous scarring at the venous puncture site but no sign of collection around the fistula nor thrombophlebitis.

Antibiotics were changed to post-dialysis cefazolin (schedule 2–2-3 grams) for a total duration of 6 weeks. She had a good clinical and biological evolution (CRP dropped to 0.6 mg/L). Follow-up blood cultures were negative. The patient pursued HD sessions three times weekly through her left forearm AVF.

**Figure 2:**
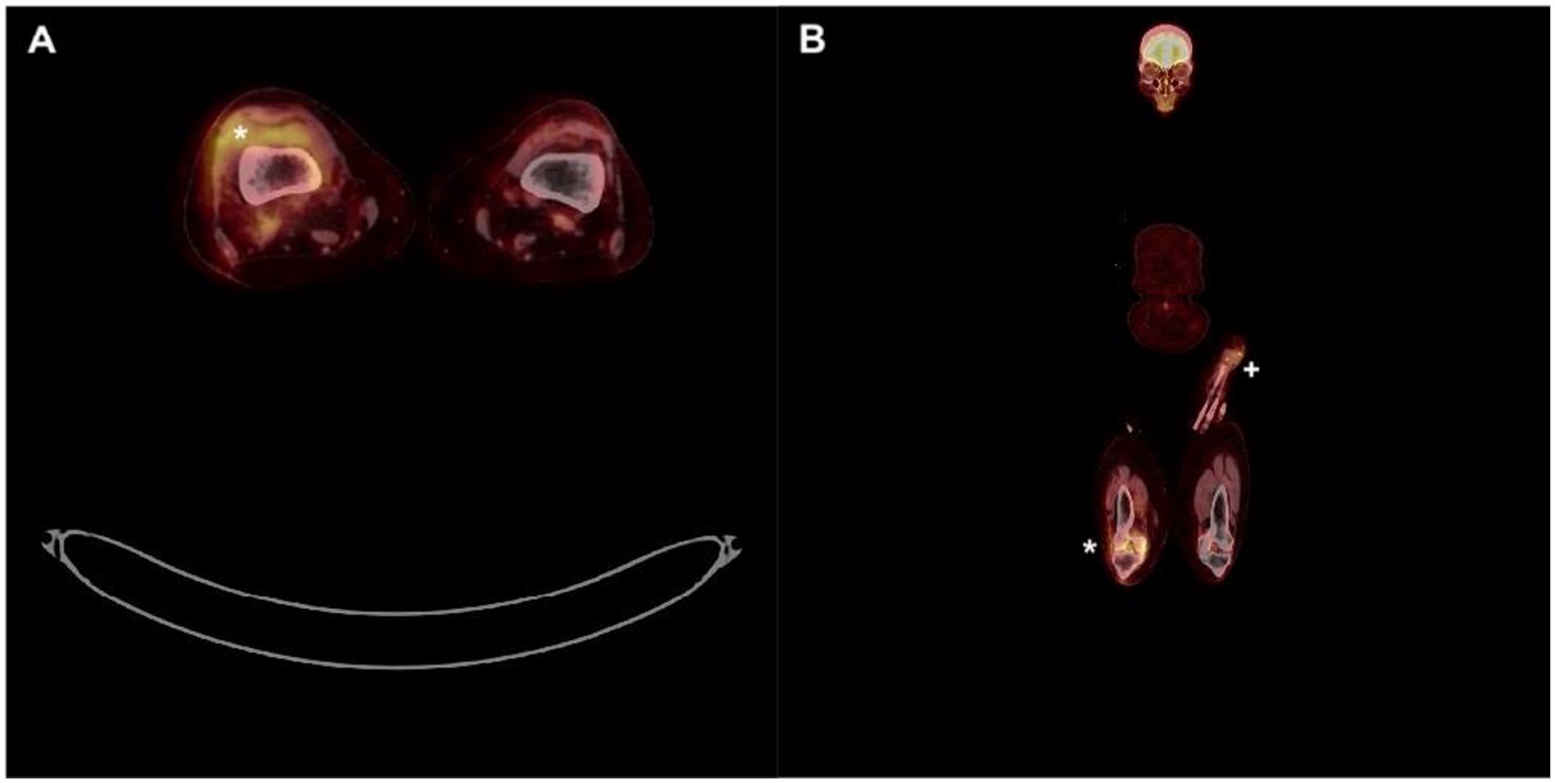
Full body 18F-FDG PET-CT. (A) Hypermetabolic synovitis of the right knee (*). (B) Focal hypermetabolic activity in the left forearm fistula area (+) as well as right knee (*).

Four months later, the patient consulted the rheumatology department with a recurrent right knee arthritis. Blood test showed an increased CRP of 96.8 mg/L, total WBC 4490/μL including 2,860 neutrophiles. Cytology from effusion of the right knee showed 6.200 RBC/μL and 183.772 WBC/μL including 87% neutrophils, 8% monocytes, 5% lymphocytes. Aerobic and anaerobic cultures from effusion of the right knee were negative but PCR 16S rRNA identified *Staphylococcus lugdunensis*. The day after admission she benefited from arthroscopic drainage and synovial biopsies were performed: bacterial cultures came back positive for *S. lugdunensis* with an identical antibiogram as during the first episode. Endocarditis was ruled out once again. Full body 18F-FDG PET-CT (Figure 2A) showed a hypermetabolic synovitis of the right knee (SUVmax 5.0). In addition, a discrete focal hypermetabolic activity in the left forearm fistula area (SUVmax 2.9) was revealed, with no visible peripheral soft tissue infiltration (Figure 2B). The hypothesis of the infection of the fistula by repeated punctures (recurrent cannulation) as entry point was evoked.

Initial treatment consisted of ceftriaxone followed by flucloxacillin and then oral combination of ciprofloxacin (500 mg daily) together with trimethoprim-sulfamethoxazole (400/80 mg twice a day) for a total duration of 12 weeks. The patient evolved well and remained asymptomatic, more than 6 months after antibiotic cessation.

## Discussion

Multiple cases of severe *S. lugdunensis* infections in the course of hemodialysis have been previously reported. In most of these patients, the presumed portal of entry is the vascular access device (9, 10). In our case, the patient had neither a foreign body nor a dialysis catheter. The main hypothesis of the entry point is repeated skin penetration through recurrent cannulation of the fistula for the dialysis access.

In a retrospective study of 36 cases of *S. lugdunensis* bacteriemia, seven patients (19%) were on hemodialysis (11). Although arteriovenous fistulas are less frequently associated with infections compared to dialysis catheters placed in the internal jugular vein, multiple cases of disseminated *S. lugdunensis* infections on hemodialysis patients with AVF have been described, including bacteriemia and endocarditis (12, 13).

The buttonhole technique consists in creating a permanent puncture site, by using a sharp needle in the same location for cannulation during each dialysis session until the creation of a tract of scar tissue. This allows for easier and more consistent needle insertion. The buttonhole cannulation is associated with less bleeding complication and aneurysm formation. However, this technic appears to be associated with an increased infectious risk (14). Constant observance of reinforced hygiene protocols by trained staff and central coordination can significantly reduce the infectious risk associated with buttonhole cannulation (14). Regular assessment and monitoring of the access site are necessary to detect any signs of complications. AVF has been proposed as a potential source for *S. lugdunensis* infections even without localized signs of infection as in our patient. The buttonhole method was proposed as a major contributor to the development and persistence of bacteremia (13). Numerous cases of *S. lugdunensis* peritonitis have been published in the context of peritoneal dialysis as well, showcasing the burden of *S. lugdunensis* infections among patients undergoing dialysis (15).

In these patients, disseminated infections are feared as *S. lugdunensis* bacteriemia are frequently associated with endocarditis. In a retrospective study of 28 cases of patients with positive *S. lugdunensis* blood culture, 13 (46%) patients had endocarditis from which 85% underwent cardiac surgery and 23% of them died (16). Fifteen patients (54%) without endocarditis had no complications related to the bacteriemia. Among these patients, 73% had an indwelling medical device. In another retrospective study of 21 cases of *S. lugdunensis* bacteriemia, among those who appeared to have clinically significant bacteriemia, five patients had catheter-related infections. Among these five patients, three were on hemodialysis (17).

However, the clinical implications of single *S. lugdunensis* blood culture positivity remain unclear. A retrospective study was published about clinical outcomes in 41 patients with single or multiple positive blood cultures (18). Overall 30-day mortality rates were 13.3% for single-set patients and 18.2% for multiple-set patients, but 90-day mortality rates were 36.7% for single-set and 18.2% for multiple-set patients. In that study, five patients with a single positive blood culture, who did not meet the criteria for true bacteriemia, and who did not receive antibiotic therapy, were followed up and evolved well. This indicates that in some low-risk clinical situations, single *S. lugdunensis* blood culture may represent contamination or low bacteriemia that might be considered clinically insignificant. Proposed criteria to better identify such patients include the absence of an apparent source of infection, time between culture collection and positivity, a low Pitt bacteriemia score (based on body temperature, hypotension, altered mental status, mechanical ventilation and cardiac arrest) and the SIRS criteria (based on body temperature, heart and respiratory rates, total WBC count) (18).

Because native joint *S. lugdunensis* septic arthritis are uncommon, we conducted a review of the literature (Table 1). We found eight previously published cases on PubMed (19–26). There were six males and two females, the median age was 54.2 years. There were seven cases of knee arthritis, one hip and one shoulder. Underlying factors such as IV drug abuse, immunosuppressive medications and immune disorders were identified most of the times. One of the eight patients was on hemodialysis as well but we found no details on the assessment of underlying bacteriemia or catheter infection. Interestingly, six patients had suffered from joint conditions before the episode of septic arthritis: three had rheumatoid arthritis, one had systematic lupus erythematosus, two had underlying osteoarthritis of the infected joint, and one had septic arthritis in the past. Regarding outcomes, two patients underwent arthroscopy washout, one had open arthrotomy debridement, one had total knee replacement and one patient had significant joint damage. Two patients were diagnosed with associated discitis and vertebral osteomyelitis. One of these patients died of acute respiratory and circulatory failure in the course of the infection. He was the only patient among the eight to be diagnosed with infectious endocarditis. Total antibiotic duration ranged from 5 to 35 weeks (two not specified). Most patients were treated with long-term IV cefazolin. Two were treated with nafcillin and two others with flucloxacillin. Only one was oxacillin-resistant and was treated with vancomycin followed by prolonged oral clindamycin.

**Table 1:** Review of the literature of native joint *S. lugdunensis* septic arthritis.

Age (years)	Sex	Year	Localisation	Antibiotherapy	Total duration	Outcomes	Risk factor	Reference
79	Male	2000	Right knee	Initial episode: IV cloxacillin (6 days) then oral flucloxacillin	35 weeks	Total replacement of the right knee, septic arthritis of the left-prosthetic knee, vertebral osteomyelitis, aortic and mitral endocarditis, death	Rheumatoid arthritis under corticosteroids and methotrexate	(19)
57	Female	2010	Right knee	Doxycycline (4 days) then IV cefazolin - gentamicin (17 days) then IV cefazolin + oral rifampicin	7 weeks	Arthroscopy of the right knee (2 times): debridement and lavage	Seronegative rheumatoid arthritis under prednisone (10 mg/day), severe osteoarthritis of the knees, type 2 diabetes	(20)
74	Male	2014	Right knee	IV flucloxacillin and sodium fusidate	16 weeks	L5 extensive epidural abscess with L2-L5 discitis and vertebral osteomyelitis. Knee wash-out and conservative management	Rheumatoid arthritis on etanercept (50 mg/week) and prednisolone (7 mg/day)	(21)
67	Male	2016	Left knee	Nafcillin 4 weeks then vancomycin-rifampicin then doxycycline	Not specified	Open medial parapatellar arthrotomy with irrigation and debridement	Left knee osteoarthritis	(22)
28	Male	2017	Right knee	Oxacillin-R: vancomycin (4 days) then clindamycin	5 weeks	Not specified	Not found	(23)
62	Male	2018	Right hip	IV vancomycin, piperacillin/tazobactam, nafcillin	6 weeks	Not specified	Prior IV heroin use, right hip MRSA septic arthritis (6 weeks before)	(24)
57	Female	2018	Left shoulder, left knee	IV cefazolin	Not specified	Not specified	SLE treated with hydroxychloroquine 200 mg and prednisone 5 mg daily, end-stage renal disease under hemodialysis, common variable immune-deficiency	(25)
10	Male	2023	Left knee	IV cefazolin, oral cephalexin	7 weeks	Chronic joint damages (fluid accumulation, thickening of the synovial membrane). Unsuccessful arthrocentesis.	Not found	(26)

IV, intravenous; L2/L5, lumbar vertebra n°2/5; Mg, milligrams; MRSA, methicillin-resistant *Staphylococcus aureus*. SLE, systemic lupus erythematosus. –R, resistant.

*Staphylococcus lugdunensis* is considered to be sensitive to most antimicrobial agents, including cefazolin, daptomycin, linezolid, moxifloxacin, nafcillin, rifampicin, quinupristin-dalfopristin, tetracycline, trimethoprim-sulfamethoxazole and vancomycin (27). Mechanisms of antibiotic therapy failure include biofilm formation (8). In a recent study by de Oliveira et al. (28), *S. lugdunensis* isolates did not show *in vitro* resistance to oxacillin, vancomycin, erythromycin, gentamicin, linezolid and sulfamethoxazole-trimethoprim. In that study, the presence of a biofilm was associated with a small increase in the MIC for linezolid and vancomycin, but did not cause resistance to these antibiotics, which was the case in Frank et al. study (27). Vancomycin was not bactericidal against 93% of *S. lugdunensis* isolates growing in biofilm, thereby suggesting vancomycin tolerance in this species. Moxifloxacin is one of the most efficient agents against *S. lugdunensis* biofilms (27). Resistance to beta-lactams and macrolides have been reported in the presence of a biofilm but remain infrequent (28).

The total recommended duration of antibiotic therapy for *S. lugdunensis* septic arthritis is at least 6 weeks according to the guidelines from the French Infectious Diseases Society (SPLIF) (29). Treatment and management should be similar to that of *S. aureus* septic arthritis. IV cefazolin or penicillin such as cloxacillin and oxacillin are the reference for the initial treatment of methicillin-sensitive strains. In the case of methicillin-resistant staphylococcus, daptomycin in monotherapy is recommended as first-line therapy with vancomycin and teicoplanin as alternatives. In our case, a first 6-week regimen of IV cefazolin was completed and led to remission. However, to treat the recurrence of *S. lugdunensis* septic arthritis, a second therapy of a 12-week oral combination of ciprofloxacin and trimethoprim-sulfamethoxazole was proposed and led to clinical and biological remission.

## Conclusion

This case report and review of the literature showcase the virulent behavior of *S. lugdunensis* and its ability to cause septic arthritis of native joints, outside the well-known context of prosthetic joint infections or arthroscopy-related joint infections.

*S. lugdunensis* is responsible for a wide range of infections in humans and should be given full consideration when isolated in bacterial cultures. Due to its aggressive behavior, some authors have drawn a parallel with *S. aureus* in terms of therapeutic management. However, it is important to bear in mind that in some cases, the identification of *S. lugdunensis* may represent contamination or be of minor clinical significance. This case also highlights the burden of disseminated and relapsing *S. lugdunensis* infections among dialysis patients. It emphasizes the need for a comprehensive and in-depth disease assessment as well as the need for adapted and extensive antibiotic therapies. Finally, it is crucial to ensure compliance with good practice guidelines for the prevention of vascular access infections in such fragile patients.

## Data Availability

The original contributions presented in the study are included in the article/supplementary material, further inquiries can be directed to the corresponding author/s.
